# Supplementary material for: Genetic Ancestry and Self-Reported “Skin Color/Race” in the Urban Admixed Population of São Paulo City, Brazil
Source: Genes (Basel). 2024 Jul 13;15(7):917. doi: 10.3390/genes15070917 (PMC11276533; doi:10.3390/genes15070917)
Supplement: Supplementary file 1 [file genes-15-00917-s001.zip › genes-3086569-supplementary.pdf]

GENETIC ANCESTRY AND SELF-REPORTED "SKIN COLOR/RACE" IN THE URBAN ADMIXED POPULATION OF SÃO PAULO CITY, BRAZIL. - SUPPLEMENTARY INFORMATION

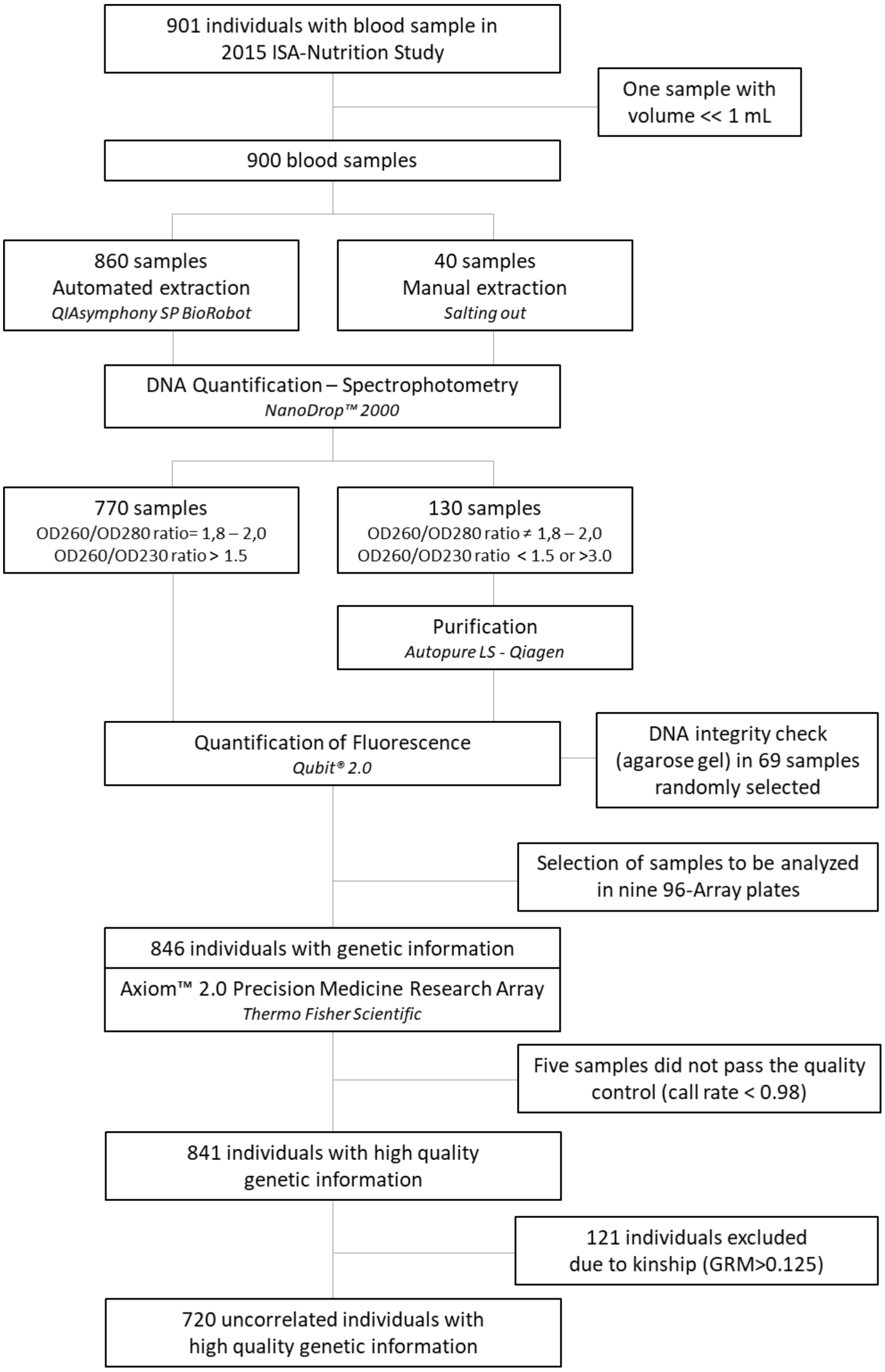

Supplementary Figure S1: Sample flowchart with details about the analysis performed.

Table S1: SNPs pruning for quality control of ancestry analysis of 720 unrelated individuals, 2015 ISA-Nutrition.

Filter	SNPs excluded	SNPs remaining
Total SNPs (Axiom™ 2.0 Precision Medicine Research Array)	NA	920,745
Recommended (Best Practices Genotyping Analysis, Thermo Fisher Scientific)	47,568	873,177
SNPs with rs notation	35,084	838,093
Autosomal	32,875	805,218
Minor Allele Frequency (<0.0001)	72,634	732,584
Hardy-Weinberg equilibrium (p<0.000001)	731	731,853
HLA-Palindromics region	461	731,392
high-LD-regions-hg19-GRCh37	20,305	711,087
LD r2>0.5 in a 50b window, shift step=5	293,895	417,192
SNPs matching ISA and 1KGP projects	178,376	238,816
Multiallelic SNPs	12,470	226,346

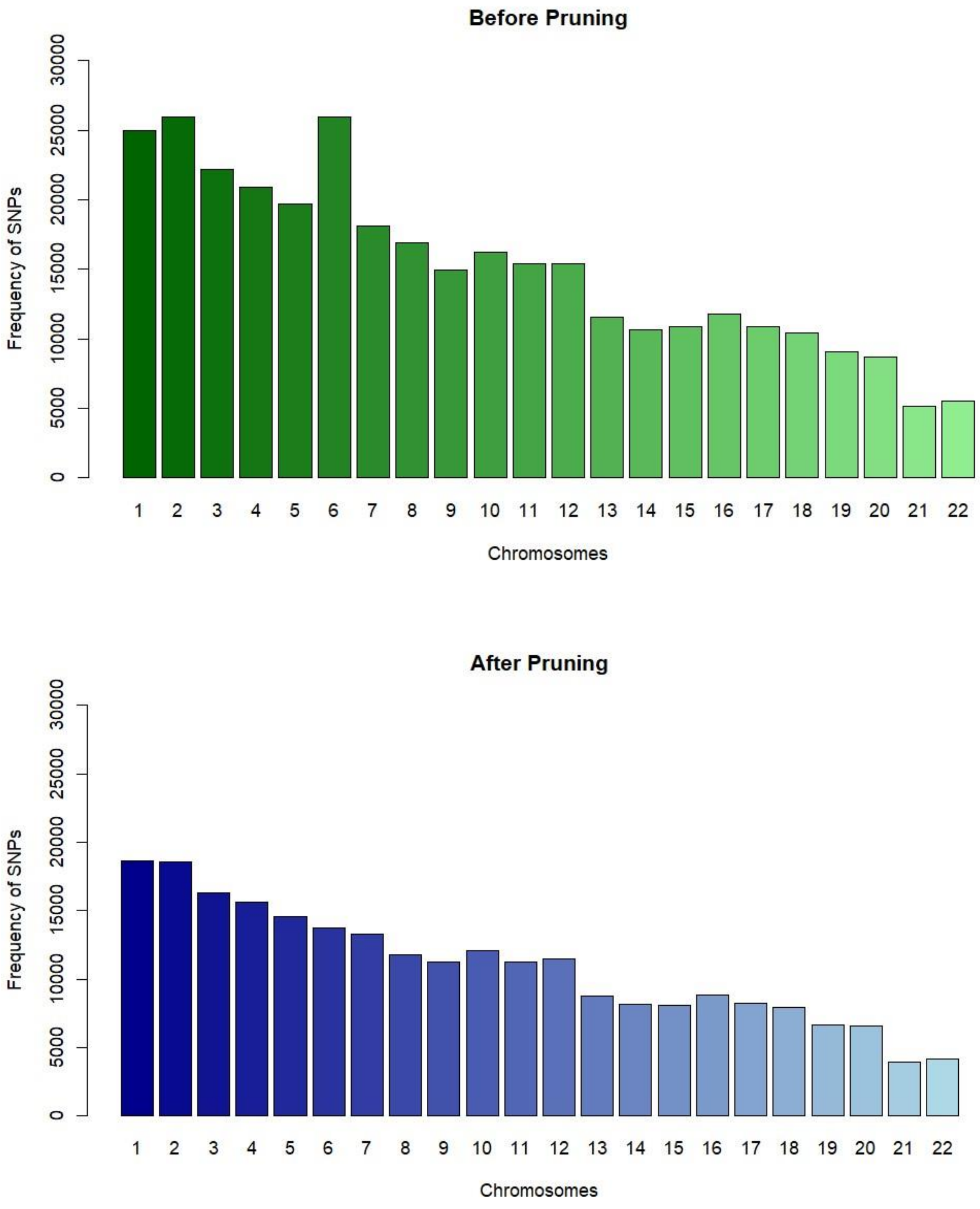

Supplementary Figure S2: Frequency of SNPs by chromosome before (in green) and after (in blue) pruning.

Table S2: Characteristics of 2015 ISA-Nutrition population according to self-reported "skin color/race" (n=841). São Paulo, 2015.

	2015 ISA-Nutrition		Self-reported color-race												p-value[a]
			Black		Indigenous		Mixed		White		Yellow		Not Answered		
	n	%	n	%	n	%	n	%	n	%	n	%	n	%	
**Total Population**	841	100	84	10.0	2	0.24	309	36.7	422	50.2	13	1.55	11	1.31	
Age group															
**Adolescent (12 |-- 20y)**	250	29.7	28	33.3	0	0	114	36.9	103	24.4	2	15.4	3	27.3	
**Adult (20 |-- 60y)**	290	34.5	29	34.5	1	50.0	108	34.9	148	35.1	3	23.1	1	9.09	
**Older adult (60 |-- 94y)**	301	35.8	27	32.1	1	50.0	87	28.2	171	40.5	8	61.5	7	63.6	0.001[a]
Sex															
**Female**	418	49.7	44	52.4	0	0	142	46.0	219	51.9	7	53.8	6	54.6	
**Male**	423	50.3	40	47.6	2	100	167	54.0	203	48.1	6	46.2	5	45.4	0.248[a]
Ancestry Proportion > 50%															
**Sub-Saharan African**	70	8.3	46	54.8	0	0	22	7.1	0	0	0	0	2	18.2	<0.001[b]
**Native American**	23	2.7	0	0	0	0	4	1.3	7	1.7	11	84.6	1	9.1	0.675[b]
**European**	674	80.1	15	17.9	1	50.0	248	80.3	400	94.8	2	15.4	8	72.7	<0.001[a]
Ancestry Proportion > 0%															
**Sub-Saharan African**	708	84.2	84	100	2	100	302	97.7	300	71.1	11	84.6	9	81.8	<0.001[b]
**Native American**	701	83.4	79	94.1	2	100	294	95.2	305	72.3	13	100	8	72.7	<0.001[a]
**European**	825	98.1	83	98.8	2	100	308	99.7	420	99.5	2	15.4	10	90.9	0.600[a]
Ancestry Proportion (%)	**Median**	**IQR**	**Median**	**IQR**	**Median**	**IQR**	**Median**	**IQR**	**Median**	**IQR**	**Median**	**IQR**	**Median**	**IQR**	
**Sub-Saharan African**	18.6	5.6 - 32.0	55.7	42.5 - 70.6	23.8	19.4 - 28.2	27.3	19.3 - 36.4	8.3	0 - 17.6	3.4	3.3 - 3.8	10.1	1.7 - 13.4	0.0001[c]
**Native American**	6.9	2.0 - 11.8	6.1	2.9 - 10.9	23.4	6.9 - 39.9	9.0	5.0 - 12.9	4.70	0 - 10.2	96.6	96.2 - 96.7	1.3	0 - 11.6	0.0001[c]
**European**	69.7	55.4 - 87.0	36.4	21.9 - 48.1	52.7	40.6 - 64.9	61.6	53.4 - 71.1	84.5	71.5 - 98.9	0	0 - 0	79.9	39.0 - 98.1	0.0001[c]

[a]p-value for Chi squared test for black, mixed and white only.
[b]p-value for Fisher's exact test for black, mixed and white only.
[c]Kruskal–Wallis equality-of-populations rank test for black, mixed and white only

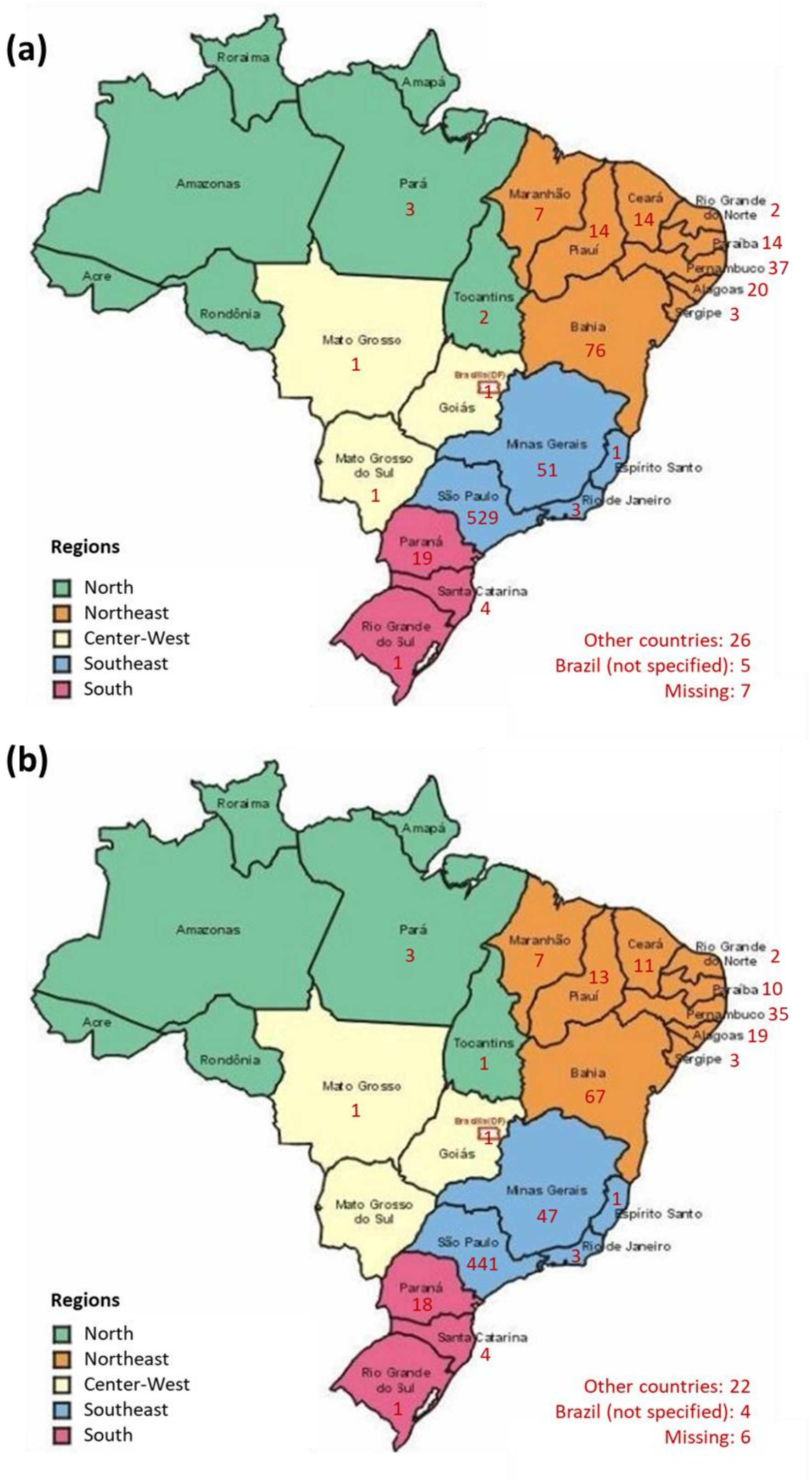

Supplementary Figure S3: Distribution of the individuals (absolute numbers) according to the place of birth in the map of Brazil for each state in the five geographical regions for (a) ISA total sample, N = 841, and (b) ISA unrelated sample, N = 720. "Other countries" refers to any country other than Brazil (the specific country names were not reported in the survey questionnaire). "Missing" indicates that the information was not provided by the respondent.

Table S3: Self-reported skin color-race as Black (N=84), Mixed (N=309), and White (N=422) according to quartiles of individual Sub-Saharan African (AFR), Native American (AMR) and European (EUR) ancestry for the total ISA sample. São Paulo, 2015.

	**Quartiles of AFR ancestry**											
	**1rst**		**2nd**		**3rd**		**4th**		**Median Regression Model**		**0.75 Regression Model**	
**Self-reported Skin Color-race**	**N**	**%**	**N**	**%**	**N**	**%**	**N**	**%**	**β (95%CI)**	**p-value**	**β (95%CI)**	**p-value**
**Black**	0	0	2	2.4	4	4.8	78	92.9	ref		ref	
**Mixed**	20	6.5	54	17.5	130	42.1	105	34.0	−0.26 (−0.30; −0.23)	<0.001	−0.36 (−0.42; −0. 30)	<0.001
**White**	184	43.6	148	35.1	70	16.6	20	4.7	−0.45 (−0.49; −0.41)	<0.001	−0.54 (−0.60; −0.48)	<0.001
										PseudoR^2=0.35		PseudoR^2=0.35
	**Quartiles of AMR ancestry**											
	**1rst**		**2nd**		**3rd**		**4th**		**Median Regression Model**		**0.75 Regression Model**	
	**N**	**%**	**N**	**%**	**N**	**%**	**N**	**%**	**β (95%CI)**	**p-value**	**β (95%CI)**	**p-value**
**Black**	14	16.7	32	38.1	21	25.0	17	20.2	ref	p-value	ref	
**Mixed**	32	10.4	78	25.2	99	32.0	100	32.4	0.03 (0.01; 0.05)	0.009	0.02 (−0.01; 0.05)	0.164
**White**	158	37.4	94	22.3	84	19.9	86	20.4	−0.01 (−0.03; 0.01)	0.212	−0.01 (−0.04; 0.02)	0.508
										PseudoR^2=0.06		PseudoR^2=0.02
	**Quartiles of EUR ancestry**											
	**1rst**		**2nd**		**3rd**		**4th**		**Median Regression Model**		**0.75 Regression Model**	
	**N**	**%**	**N**	**%**	**N**	**%**	**N**	**%**	**β (95%CI)**	**p-value**	**β (95%CI)**	**p-value**
**Black**	73	86.9	9	10.7	2	2.4	0	0	ref	p-value	ref	
**Mixed**	100	32.4	126	40.8	65	21.0	18	5.8	0.26 (0.21; 0.32)	<0.001	0.23 (0.20; 0.27)	<0.001
**White**	31	7.4	69	16.4	137	32.5	185	43.8	0.49 (0.43; 0.54)	<0.001	0.45 (0.42; 0.49)	<0.001
										PseudoR^2=0.30		PseudoR^2=0.35

Quantile regression adjusted for age.
β is the coefficient model, and 95%CI is the Confidence Interval of 95%.

Table S4: Genetic variance explained by the first six components in the Principal Component Analysis for the ISA + 1KGP (PC1 + PC2 ~ 84.8%) and exclusively for the ISA sample (PC1 + PC2 ~ 35.8%.). São Paulo, 2015.

**ISA sample**						
	PC 1	PC 2	PC 3	PC 4	PC 5	PC 6
Min.	−0.1339	−0.2387	−0.2731	−0.5437	−0.4959	−0.0785
1st Qu.	−0.0189	−0.0046	−0.0111	−0.0016	−0.0007	−0.0065
Median	0.0045	0.0065	−0.0016	0.0021	0.0028	−0.0015
Mean	0	0	0	0	0	0
3rd Qu.	0.0248	0.016	0.011	0.0062	0.0062	0.0026
Max.	0.049	0.0349	0.259	0.0849	0.0412	0.5267
**ISA + 1KGP sample**						
	PC 1	PC 2	PC 3	PC 4	PC 5	PC 6
Min.	−0.0205	−0.0259	−0.1482	−0.0577	−0.1028	−0.069
1st Qu.	−0.0145	−0.0206	0.0008	−0.0027	−0.0057	−0.0071
Median	−0.0114	−0.0008	0.003	−0.0005	0.0023	−0.0005
Mean	0	0	0	0	0	0
3rd Qu.	0.0212	0.0077	0.0093	0.0017	0.0132	0.0072
Max.	0.036	0.0338	0.0154	0.0756	0.0471	0.0666

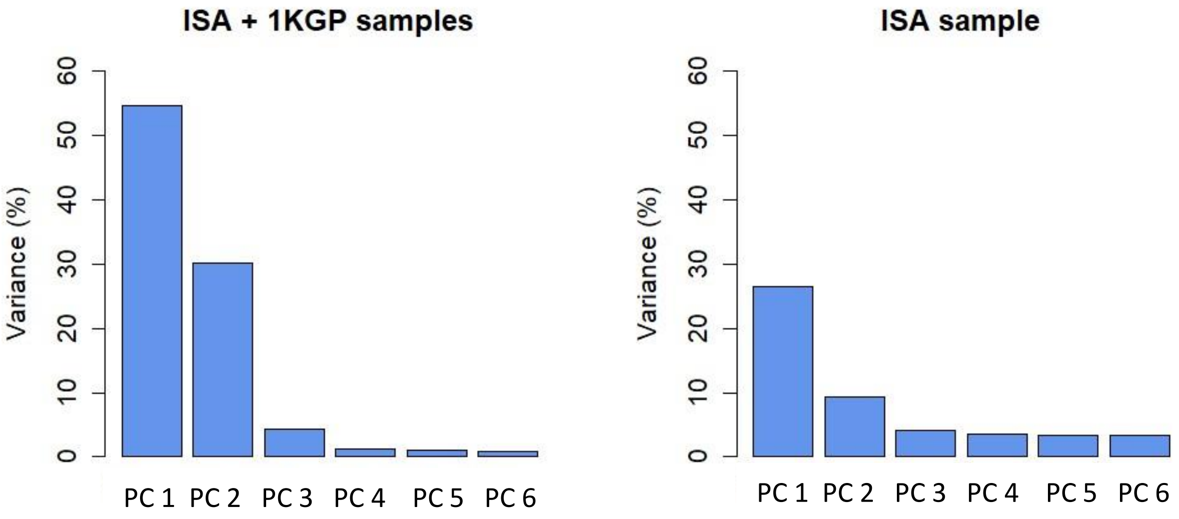

Supplementary Figure S4: Genetic variance explained by the first six components in the Principal Component Analysis for the ISA + 1KGP (PC 1 + PC 2 ~ 84.8%) and exclusively for the ISA sample (PC 1 + PC 2 ~ 35.8%.).

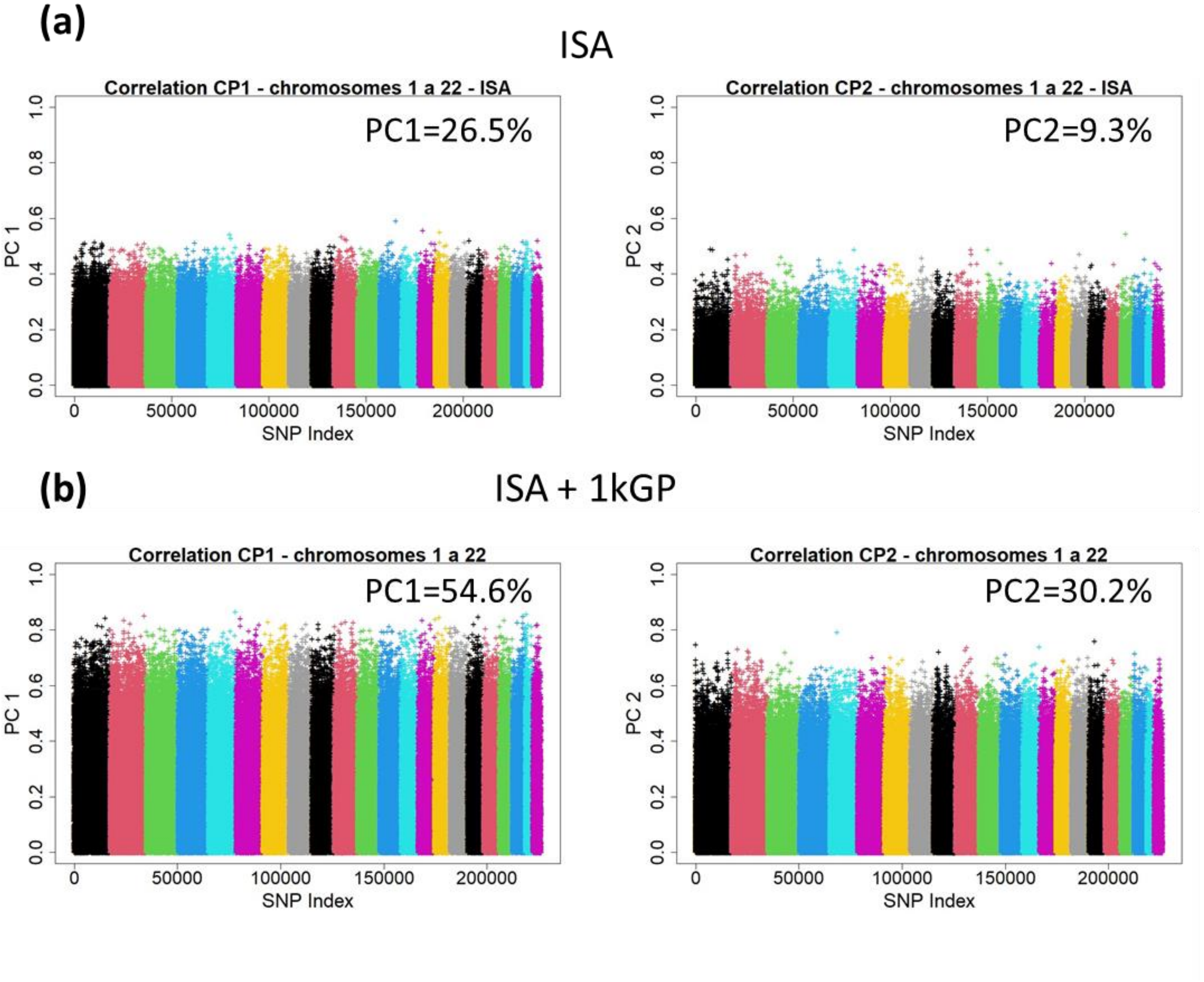

Supplementary Figure S5: Correlation of the first and second Principal Components (PCs) per chromosome (1 to 22) in ISA sample (a) and in ISA + 1KGP samples (b).

(a)

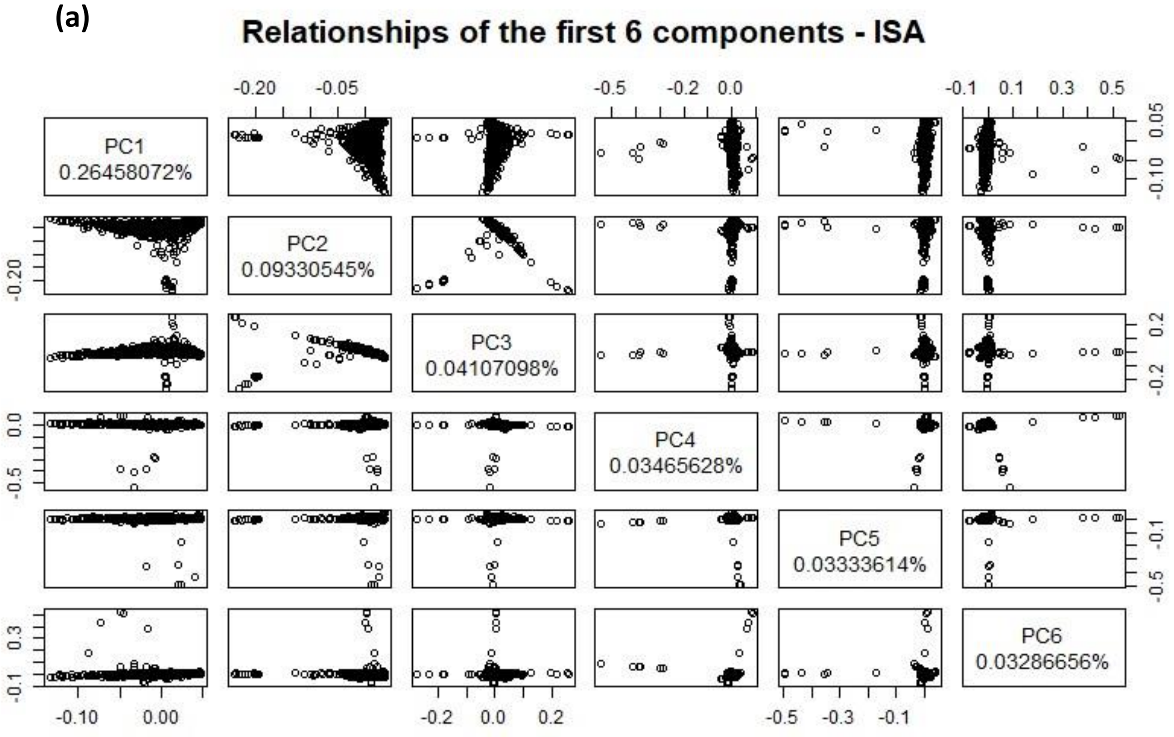

(b)

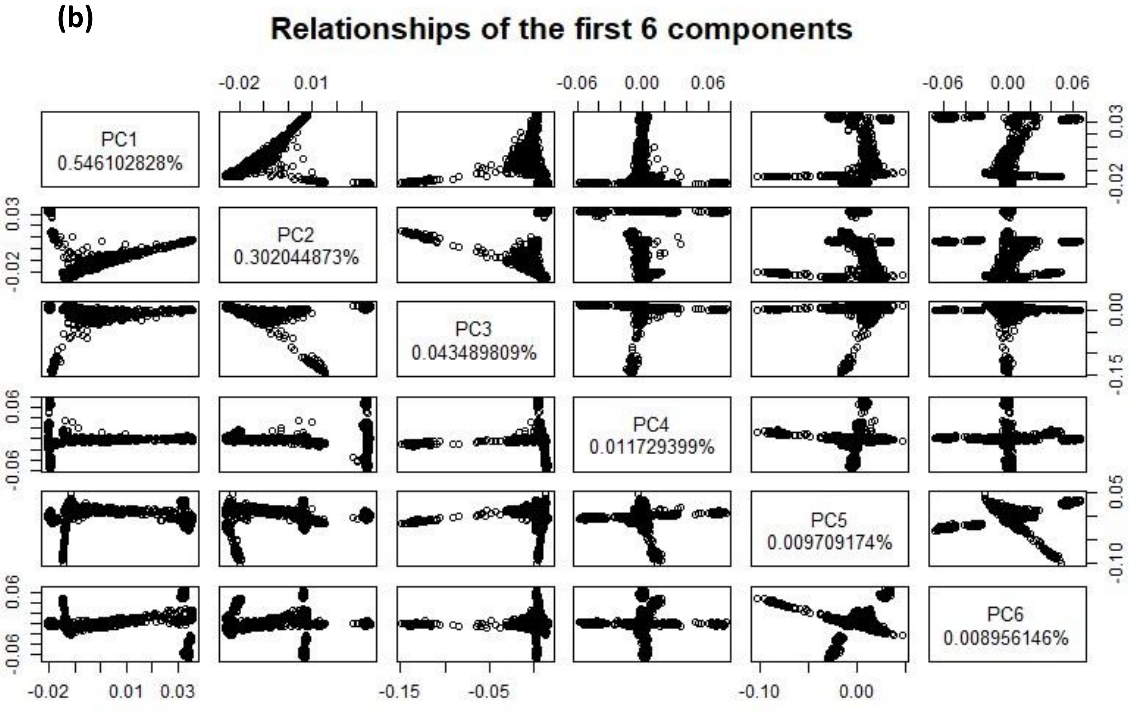

Supplementary Figure S6: Correlation between the first six Principal Components in the PCA in ISA sample (a) and in ISA + 1KGP samples (b).

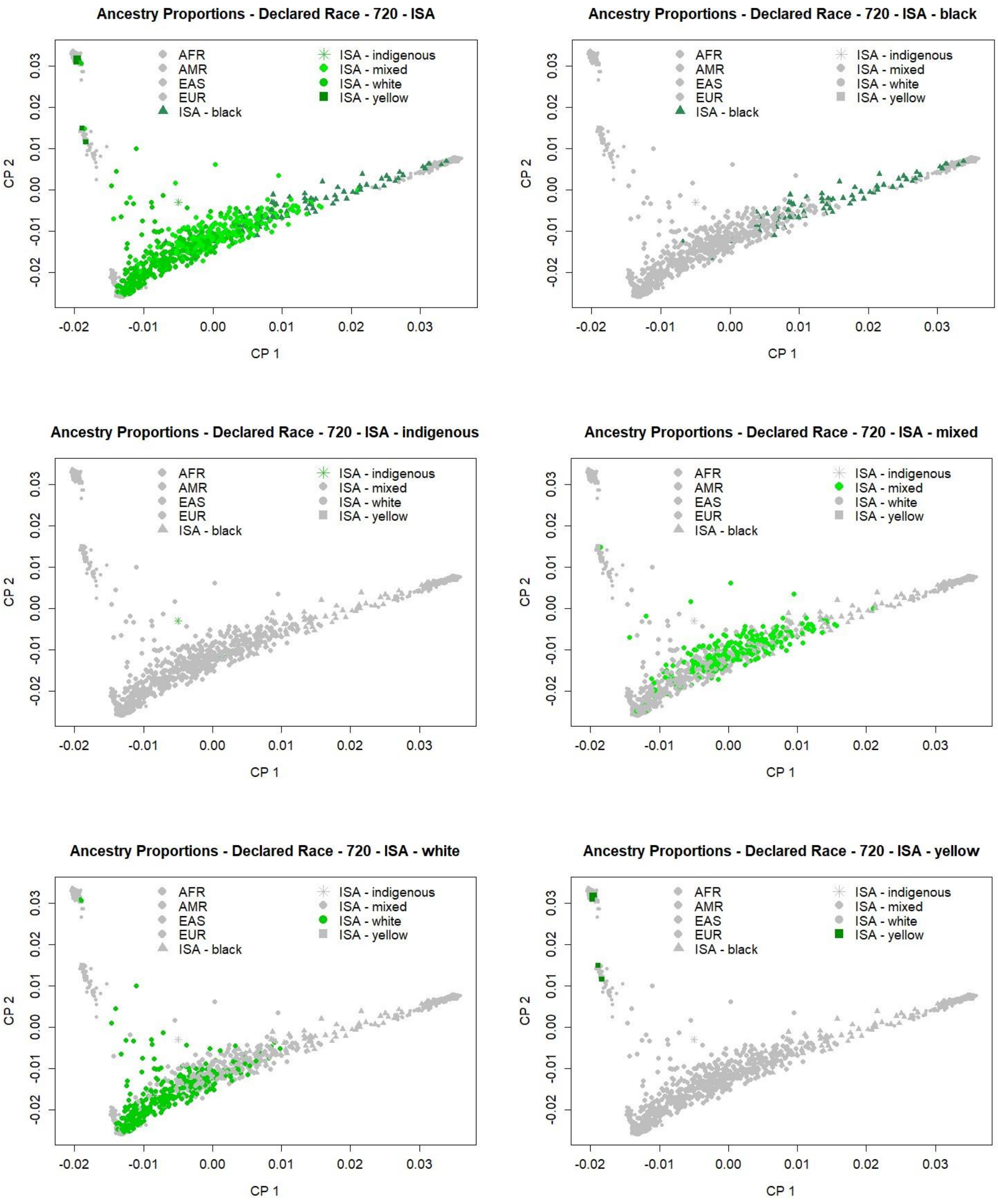

Supplementary Figure S7: Scatterplot showing the first two principal components for the ISA-Nutrition sample according to 1KGP reference populations, with the self-reported “skin color/race” displayed separately for each category for easier visualization of the results.
